# Feasibility of a computer-assisted alcohol screening, brief intervention and referral to treatment program for DWI offenders

**DOI:** 10.1186/s13722-015-0046-1

**Published:** 2015-11-09

**Authors:** Jillian Mullen, Stacy R. Ryan, Charles W. Mathias, Donald M. Dougherty

**Affiliations:** Psychiatry Department, The University of Texas Health Science Center at San Antonio, NRLC MC 7793, 7703 Floyd Curl Drive, San Antonio, TX 78229-3900 USA

**Keywords:** Driving while intoxicated, Computer-assisted, Alcohol, Brief intervention, SBIRT

## Abstract

**Background:**

Alcohol use patterns that are hazardous for one’s health is prevalent among DWI (driving while intoxicated) offenders and is a key predictor of recidivism. The aim of this program evaluation was to determine the feasibility and usability of implementing a computer-assisted screening, brief intervention and referral to treatment (SBIRT) program for DWI offenders to enable the identification of those in need of treatment services soon after arrest. Our treatment program consisted of a web-based, self-guided screening tool for assessing alcohol use patterns and generating a personalized feedback report that is then used to deliver a brief motivational intervention and if needed, a referral to treatment.

**Methods:**

Between August and November 2014, all DWI offenders attending orientation for pre-trial supervision were assessed for eligibility. Of the 129 eligible offenders, 53.5 percent enrolled and the first 50 were asked to complete a usability and satisfaction questionnaire.

**Results:**

The results demonstrated that the majority of those screened reported at-risk alcohol use patterns requiring referral to treatment. Clients reported high ratings of usability and satisfaction with the screening tool and personalized feedback report, which did not significantly differ depending on alcohol use patterns. There were relatively few technical difficulties, and the majority of clients reported high levels of satisfaction with the overall SBIRT program.

**Conclusion:**

Results of this program evaluation suggest that computer-assisted SBIRT may be successfully implemented within the criminal justice system to DWI offenders soon after arrest; however, further research is required to examine its effects on treatment utilization and recidivism.

## Background

Problematic patterns of alcohol consumption are highly prevalent among DWI (driving while intoxicated) offenders [1, 2], which increases their odds of recidivism [3]. Indeed, those who recidivate tend to show more frequent and heavier drinking patterns and are more likely to meet clinical diagnostic criteria for an alcohol use disorder [2, 4, 5]. Despite the high prevalence of alcohol use disorders among DWI offenders, a substantial proportion are not receiving treatment; further, those reporting no private health insurance and low income appear to be the most vulnerable to unmet treatment needs [6]. Taken together, these findings suggest that DWI offenders represent a clinically underserved population and demonstrate the need for the identification and treatment of problematic alcohol use to reduce recidivism among this population and enhance public safety.

Currently, screening for alcohol use problems and referral to treatment processes within the criminal justice system varies widely between counties and states [7, 8]. For most DWI offenders, screening is not initiated until after adjudication, which can take months or even years [9], thereby delaying the identification of those in need of treatment. One possibility for changing this would be to conduct alcohol use screening during pre-trial services. Pre-trial services orientation sessions may provide an opportune moment to engage offenders in their own treatment and recovery process. Pre-trial services provide supervision of offenders prior to adjudication, and this supervision process starts soon after release from custody for the offense. Considering the high rates of recidivism and the cost to public safety, screening DWI offenders for patterns of problematic alcohol use soon after arrest might help to prevent recidivism.

Screening, brief intervention and referral to treatment (SBIRT) is a short, cost-effective approach that can be delivered by nonclinical staff to identify problematic alcohol use patterns, intervene, and help guide offenders who need treatment to more specialized services [10, 11]. SBIRT is implemented primarily in medical settings. There is an extensive body of literature demonstrating that it is feasible to integrate screening and brief intervention services within broader, existing healthcare systems (i.e., primary care clinics and emergency departments) using nonclinical staff; these services can effectively reduce problematic alcohol consumption and related consequences [12–14]. The SBIRT model consists of using standardized screening measures to assess an individual’s alcohol use patterns and establish their level of risk for problems associated with use. A brief intervention, varying from education for low-risk users, brief motivational interviewing for at-risk users, and brief motivational interviewing and referral to treatment for high-risk users, is then provided. Because SBIRT is designed to be incorporated into the framework of an already-existing process to provide opportunistic screening and intervention services by nonclinical staff in nontraditional settings [15], it potentially could be implemented in other settings, such as the criminal justice system. Due to the rates of untreated alcohol problems among DWI offenders and the association between problematic patterns of alcohol use and repeat offending, SBIRT may offer a relatively low-cost intervention that could have significant impact on public safety.

As part of a treatment clinic, we are developing an SBIRT program specifically for use with DWI offenders for implementation within the criminal justice setting. In order to enhance standardization and ease-of-implementation, we developed a computer-assisted SBIRT program. Traditionally, the screening aspect of SBIRT consists of paper-and-pencil questionnaires being administered and scored by staff, which can be costly in terms of time and personnel resources. Using computers to facilitate the screening aspect of SBIRT, however, offers the potential to minimize such barriers to implementation [16]. Additionally, computer-assisted screening provides the opportunity to quickly generate personalized feedback that can be used by staff to provide a brief intervention which, compared to generic feedback, may have greater impact on behavioral change [17].

Our SBIRT program consists of a self-guided, web-based, screening tool named Motivational Alcohol Treatments to Enhance Roadway Safety (MATTERS), which assesses alcohol use characteristics and generates a personalized feedback report. The feedback report can then be used by staff to deliver a manualized brief motivational intervention and provide a referral to treatment [18]. The aim of the current program evaluation was to examine the feasibility of implementing computer-assisted SBIRT soon after arrest to DWI offenders most vulnerable to unmet treatment needs (i.e., low income and/or uninsured; [6]) during the pre-trial services phase of criminal justice involvement. We sought to: (1) examine rates of participation; (2) assess the willingness of offenders to report problematic alcohol use and symptoms of the most common co-morbid mental health disorders using the computerized screening tool; (3) examine the usability of the computerized screening tool by assessing clients’ perceptions of ease-of-use, time, aesthetics, and comprehension, as well as staff reports of assistance given to clients and technical issues; (4) examine acceptability of the personalized feedback report in terms of perceived personal value, perceived impact on alcohol use, and comprehension; and (5) assess client satisfaction with the SBIRT session.

## Methods

The information reported herein includes general statistics about the implementation of a newly developed assessment tool used by the MATTERS outpatient clinic. No personal identifiers are contained within the data, and no identifying behaviors are addressed within the assessment. The analysis of this assessment and evaluation of the MATTERS screening tool described in this paper was determined to be exempt from IRB review.

During court-mandated DWI pre-trial orientation sessions, DWI offenders most vulnerable to unmet treatment needs were referred to our SBIRT program. Clients first completed the MATTERS screening tool. A counselor, using the personalized feedback report generated from the tool, then administered a brief motivational intervention depending on the client’s level of risk and if needed, provided a referral for treatment. The SBIRT program was manual-based and developed by our clinic [18]. Upon completion of the session, clients were asked to complete a usability and satisfaction questionnaire. The procedure and each of these components are described in more detail below.

### Participants

As part of the standard court procedure, all adults arrested for DWI are scheduled to attend a pre-trial orientation session, usually within 2-weeks of their arrest. DWI pre-trial orientation sessions were held three times per week in one of the largest counties in the southwestern United States’ pre-trial services department. Clients were referred for screening from all available sessions from August 2014 to November 2014. Because this program is being developed for those most at-risk for unmet treatment needs, clients had to meet at least one of the following criteria to enter our SBIRT program: uninsured, Medicaid or Medicare eligible, low income (<$26,000 annual income, 200 % of the single individual household federal poverty threshold) [19], or legally indigent. Of the defendants who were eligible and completed our program (n = 69), the first 50 (41 men, 9 women) were asked to complete a usability and satisfaction questionnaire and are the subjects of this report.

### MATTERS screening tool

The MATTERS screening tool is a web-based program for assessing alcohol use characteristics and generating personalized feedback reports. The program takes approximately 15–20 min to complete independently by the user. This assessment focuses on gathering information that would be particularly motivating when framed within a personalized feedback report. It includes: quantity and frequency of alcohol use, as well as the corresponding estimated blood alcohol concentrations (BAC); drunk driving behavior; family history of alcohol disorders; alcohol use risk (Alcohol Use Disorders Identification Test, AUDIT) [20]; negative consequences experienced as a result of alcohol use (modified Short Inventory of Problems, SIP) [21]; estimated financial impact of DWI; and strategies that could be useful for reducing alcohol use. Based on the client’s responses, a personalized feedback report is generated consisting of text and graphics summarizing the factors assessed within different sections: typical alcohol use, personal risk, cost-of-use, and strategies to change alcohol use.

The MATTERS screening tool in this instance was completed on a Microsoft Surface™ Pro-2 tablet. In this system, clients first read introductory instructions and then worked through a series of pages to provide answers to the screening assessment. Clients were required to use the touch screen to navigate through the assessment and make their responses, as well as use a keyboard attachment when they were required to type a response. To navigate between pages, clients used the touch-screen mode to select a “next page” button marked at the bottom right-hand corner of the screen. The assessment section presented short instructions and limited the number of questions per page so that all would fit within the screen and avoid the need to scroll to complete each page. Depending on the question type, clients were required to respond by choosing the appropriate answer from a drop-down list of pre-defined responses, by checking a box, or by typing in a numerical value using the keyboard attachment.

To develop the MATTERS screening tool, we adapted the commercially available Electronic-Check-Up to Go program (e-CHUG; available from http://www.e-chug.com and http://www.3rdmilclassrooms.com), which is an evidence-based program that includes a brief assessment of alcohol use and generates a personalized feedback report tailored to college students [22]. We adapted and modified the assessment questions and feedback report content to tailor the intervention specifically to DWI offenders by providing enhanced education on BAC levels, driving while intoxicated, and money spent on alcohol (including DWI fees), as well as additional information on the negative consequences experienced as a result of alcohol use and strategies specific to reducing alcohol consumption. Our adapted version also included modified language (Flesch-Kincaid grade level 5.2 and Flesch-Kincaid Reading Ease 78.8) to ensure comprehension across a wide range of adults with varied reading abilities and modified graphics to facilitate comprehension.

### Measures

#### Usability and satisfaction

Based on previous research [23–25], a self-report usability and satisfaction questionnaire was developed to assess: (1) prior computer experience and overall comfort level; (2) perceptions of the computerized assessment; (3) perceptions of the personalized feedback report; and (4) satisfaction with the overall session. Counselors also completed a usability report.

#### Prior computer experience

To assess prior computer experience, clients were asked: (a) “*How often do you use a computer?*” responding “*never, monthly, weekly,* or *daily,*” and (b) “*How comfortable are you using computers?*” responding on a 7-point Likert scale ranging from “*not at all*” to “*extremely.*”

#### Client usability report

To assess perceptions of the MATTERS screening tool, clients were asked to respond on a 7-point Likert scale ranging from “*strongly disagree*” to “*strongly agree*” on how true each statement was for them: (a) “*The computer program was easy to use.*” (b) “*The amount of time it took to complete the computerized part of the session was acceptable.*” (c) “*I like how the computer program looked.*” (d) “*The instructions were easy to understand.*” and (e) “*The questions were easy to understand.*” To assess perceptions of the personalized feedback report, clients were asked to respond on a 7-point Likert scale with appropriate anchors for each question: (a) “*Overall, how interesting was the information?*” (b) “*Overall, how personally relevant did you find the information?*” (c) “*How might this information impact your drinking?*” (d) “*Does this information increase your motivation to reduce drinking?*” (e) “*How effective do you think this would be in reducing other DWI defendants’ alcohol use?*” (f) “*Was the information presented clearly?*” and (g) “*How well could you understand the information?*” Clients were also given the option to report anything they did not like or would like to change about the screening tool, personalized feedback report or overall SBIRT session.

#### Counselor usability report

For each testing session, counselors were asked to respond on a 7-point Likert scale (ranging from “*no assistance*” to “*full assistance*”) with regards to how much assistance was provided to the client to complete the MATTERS screening tool and to provide a description of the assistance provided. Counselors were also asked to report and describe any computer issues.

#### Client satisfaction

To assess client satisfaction with the overall session, clients were asked: a) “*The amount of time taken to complete the session was acceptable,*” responding on a 7-point Likert scale ranging from “*strongly disagree*” *to* “*strongly agree;*” and b) “*How helpful was this session for you?*” responding on a 7-point Likert scale ranging from “*extremely unhelpful*” to “*extremely helpful;*” and c) to report anything that they did not like or would like to change.

#### Nonparticipation

To assess potential limitations regarding participation, a self-report questionnaire was developed to assess the characteristics (age, sex, gender, race, and ethnicity) of DWI defendants who declined to participate, as well as their reason for declining: (a) “*I have already started treatment.*” (b) “*I do not have a drinking problem.*” (c) “*I am not interested.*” and (d) “*other,*” with space to elaborate on their reason. Those who declined taking part in our SBIRT program were also asked to complete the AUDIT for purposes of comparison to SBIRT completers.

### Procedure

All DWI defendants attending the DWI pre-trial orientation session were required by the court to meet individually with a pre-trial officer to discuss conditions of their pre-trial supervision. During this interview, pre-trial officers assessed the eligibility (see “Participants”) of the defendants for our program. Eligible defendants were escorted by a clinic staff member to a waiting area to be seen by a counselor and given a brief description of the service. Those who provided verbal consent were given the option to complete the session in a private interview room within pre-trial services with the first available counselor or to provide their contact details to arrange a later appointment at our clinic. The first 20 defendants to decline were asked to complete a short nonparticipation questionnaire regarding their reasons for declining and the AUDIT. Defendants received $10 for completion of the questionnaire.

Each session was conducted by a Licensed Professional Counselor (LPC). Although SBIRT can be delivered by nonclinical staff, LPC-level counselors were used for feasibility testing because the MATTERS clinic incorporates the provision of extended services. All sessions began immediately after clients signed a written consent-to-treatment form and agreed to be included in the use of general statistics for the assessment and evaluation of the MATTERS screening tool. Using the tablet, clients were first asked to provide demographic information (age, sex, race, ethnicity, education, and employment status) and report on their mental health status using the Primary Care Post-Traumatic Stress Disorder (PC-PTSD) screen [26], the Generalized Anxiety Disorder-2 (GAD-2) scale [27], and the Patient Health Questionnaire depression module-2 (PHQ-2) [28]. Counselors then logged the client into the MATTERS screening tool and left the interview room to allow the client to complete the assessment. Upon completion of the MATTERS screening tool, a personalized feedback report was printed for the client. Next, the counselor used this as a tool to deliver a brief motivational intervention. Depending on the AUDIT score and risk category (low risk, risky, harmful, and dependent) [29], clients were then provided a referral to treatment in the community. Clients were provided a referral list for inpatient and outpatient services consisting of free, reduced-cost, private, faith-based, and veteran options to choose from. Sessions conducted at pre-trial services (n = 38) were completed in one of four private interview rooms with glass windows overlooking the waiting area, with a white noisemaker set outside of the interview room to increase privacy. At our clinic, sessions (n = 12) were conducted in one of two private treatment rooms. Upon completion of the session, clients were approached by clinic staff to complete a usability and satisfaction questionnaire, and the counselor completed the counselor usability report. Clients received $10 for completion of the questionnaire.

## Results

### Feasibility

#### Participation rates

There were 129 DWI defendants who were eligible for referral to our SBIRT program during the measurement period. Of those eligible, 53.5 percent completed the SBIRT session, with the majority of those (71.0 %) completing it at pre-trial services. Only the first 50 clients who participated in our SBIRT program were included in the usability and satisfaction evaluation.

#### Clients

Of the first 50 clients (41 men, 9 women) to complete the SBIRT session, one client (male) was unable to complete the MATTERS screening tool due to a computer issue (described below) and instead completed a paper-and-pencil version. As a result, this client was not asked to complete the usability and satisfaction questionnaire and was only included in the description of computer issues (see below). Client characteristics are displayed in Table 1. Clients were, on average, 40.90 (SD = 11.31) years of age. The majority of clients were white men of Hispanic ethnicity with at least a high school education and full-time employment.

**Table 1:** Client characteristics (*n* = 49)

Characteristic	*n* (%)
Male	40 (82)
Race	
African American	1 (2)
White	46 (94)
Multiracial/other	2 (4)
Ethnicity, *Hispanic*	37 (76)
Education	
Less than high school	5 (10)
High school graduate	13 (27)
Partial college	28 (57)
4-year degree or more	3 (6)
Employment	
Full-time	29 (59)
Part-time	6 (12)
Unemployed	5 (10)
Disabled/retired/student/career	9 (18)
AUDIT risk level	
Low Risk	13 (27)
Risky	21 (43)
Harmful	4 (8)
Dependent	11 (22)
Mood disorder screens	
GAD-2 (score ≥3)	14 (29)
PC-PTSD (score ≥2)	10 (20)
PHQ-2 (score ≥3)	12 (25)

	M (SD)
Age	40.90 (11.31)

Scores indicated for each mood disorder screening measure suggest further assessment is required

Of the 49 DWI offenders screened using the MATTERS screening tool, 28.6 percent endorsed symptoms for generalized anxiety disorder, 20.4 percent endorsed symptoms for PTSD, and 24.5 percent endorsed symptoms for depression at levels that suggested a need for further assessment (see Table 1). Based on AUDIT scores, the majority (73 %) of clients screened were classified as “risky” or above, highlighting that the majority were willing to report using alcohol at a level that put them at risk for legal, physical, and health-related problems, and required referral for further treatment (see Table 1).

#### Defendants who declined participation

The first 20 defendants to decline treatment services (i.e., participation in the SBIRT session) were asked and agreed to complete a short questionnaire on reasons for declining. The characteristics of defendants who declined participation in the SBIRT session are presented in Table 2. Similar to defendants who completed the SBIRT session, the majority of the 20 who declined were white men of Hispanic ethnicity with a mean age of 39.08 (SD = 16.17) years. In contrast to those who participated, a larger proportion of those who declined had AUDIT scores in the low-risk category, and a lower proportion were classified as harmful or dependent. With regards to reasons for not participating, most reported that either they “did not have a drinking problem” (n = 7) or “were not interested in the session” (n = 6). Four defendants who reported “other” stated further that they declined to participate because they did not have time (n = 2), that they had to have it approved by their attorney before continuing (n = 1), or were already seeing a counselor for other issues (n = 1).

**Table 2:** Characteristics of DWI defendants (n = 20) who declined participation

Characteristic	n (%)
Male	19 (95)
Race, *Caucasian*	19 (95)
Ethnicity, *Hispanic*	15 (75)
AUDIT risk level	
Low risk	9 (45)
Risky	8 (40)
Harmful	1 (5)
Dependent	2 (10)
Reason for declining	
I have already started treatment	3 (15)
I do not have a drinking problem	7 (35)
I am not interested	6 (30)
Other	4 (20)

	*M* (*SD*)
Age	39.08 (16.07)

### Usability and satisfaction

#### Prior computer experience

The majority of clients reported previous experience using a computer (77 %), with 51 percent daily users, 22 percent weekly users, and 4 percent monthly users. Overall, clients reported a relatively high level of comfort using computers (M = 5.29, SD = 2.06 on a 7-point scale).

#### Client usability

As displayed in Table 3, the majority of clients rated the ease-of-use and appearance of the MATTERS screening tool as positive (responses were considered positive if ≥5), with mean ratings of 6.00 (SD = 1.76) and 6.25 (SD = 1.42), respectively, on the 7-point scale. The majority of clients also rated the comprehension of instructions and questions as positive, with overall mean ratings of 6.73 (SD = 0.87) and 6.38 (SD = 1.16), respectively. Acceptability ratings were similar for low risk, risky, and harmful/dependent drinkers, with no significant differences between the groups (all *p’s* > 0.7). With regards to changes that could be made to the MATTERS screening tool, three clients commented that the size of the screen/text could be bigger; one client requested that “unknown” should be one of the possible answers to family history risk for alcohol use disorders question; one client suggested that it could be quicker; and one client requested the ability to provide dates when reporting typical alcohol consumption over the past month.

**Table 3:** Percent of clients by AUDIT risk level, rating the MATTERs screening tool acceptability factors as positive

Acceptability factors	AUDIT risk category			
	Low risk (n = 13) %	Risky (n = 21) %	Harmful/dependent (n = 15) %	Total (n = 49) %
The computer program was easy to use	84.6	76.2	73.3	77.6
I like how the computer program looked	92.3	90.5	86.7	91.7
The instructions were easy to understand	92.3	100.0	86.7	95.8
The questions were easy to understand	92.3	90.5	86.7	91.7

As displayed in Table 4, the majority of clients reported that the information presented in the personalized feedback report was highly interesting and personally relevant, with mean ratings of 6.44 (SD = 1.02) and 6.38 (SD = 1.26), respectively, on the 7-point scale. In addition, the majority of clients also reported that the information would be highly likely to impact their drinking, increase their motivation to reduce drinking, and be effective in reducing other DWI defendants’ alcohol use, with mean ratings of 6.27 (SD = 1.35), 6.37 (SD = 1.32), and 6.37 (SD = 1.15), respectively. The majority of clients also rated the presentation and comprehension of the information as positive, with overall mean ratings of 6.78 (SD = 0.78) and 6.78 (SD = 0.59), respectively. Acceptability ratings were similar for low risk, risky, and harmful/dependent drinkers, with no significant differences between the groups (all *p’s* > 0.35).

**Table 4:** Percent of clients by AUDIT risk level rating the personalized feedback report acceptability factors as positive

	AUDIT risk category			
	Low risk (n = 13) *%*	Risky (n = 21) *%*	Harmful/dependent (n = 15) *%*	Total (n = 49) *%*
How interesting was the information	92.3	95.2	100.0	95.9
How personally relevant did you find the information	92.3	95.2	100.0	95.9
How might this information impact your drinking	92.3	95.2	86.7	91.8
Does this information increase your motivation to reduce drinking	92.3	90.5	100.0	93.9
How effective do you think this would be in reducing other DWI defendants alcohol use	92.3	95.2	93.3	93.9
Was this information presented clearly	92.3	95.2	100.0	95.9
How well could you understand the information	92.3	100.0	100.0	98.0

#### Counselor usability

On 12 out of 49 occasions (24.4 %), the counselors reported having to assist clients in completing the screening assessment. Most often (n = 9), this was due to clients’ difficulty with viewing the writing on the screen. Text size was reported as being too small for clients to read, so the counselors helped by reading the questions and recording clients’ responses. On two occasions, the counselors had to complete the screening assessment with the clients because the clients were unfamiliar with computers and had difficulty using the tablet; on one occasion, the counselor had to provide additional instruction to complete one particular section (not all questions had been answered, so the program would not progress).

All but one client who started the MATTERS screening tool were able to complete the assessment (the tablet would not register the keyboard or touch-screen presses). On five other occasions, there were minor computer or printer issues: screen froze (n = 2), printer froze (n = 2) and lost internet connection (n = 1), but these were quickly resolved by the counselor and the clients were able to complete the assessment.

#### Client satisfaction

The MATTERS screening tool took on average 20 min to complete, and the full SBIRT session lasted on average 53.97 (SD = 12.74) min. Overall, 85.7 percent of clients reported that the time taken to complete the MATTERS screening tool was acceptable (M = 6.13, SD = 1.79), and 95.9 percent reported that the time taken to complete the entire SBIRT session was acceptable, (M = 6.49, SD = 1.10). In addition, 93.9 percent reported that they found the session to be helpful (M = 6.53, SD = 1.14).

#### Location

We further assessed any potential difference in responding from those receiving SBIRT at pre-trial (n = 38) and those receiving SBIRT at the clinic (n = 12). Both groups were comparable in the range of scores for the usability and client satisfaction domains.

## Discussion

The aim of this program evaluation was to examine the feasibility of implementing computer-assisted SBIRT soon after arrest for DWI offenders, during their pre-trial services orientation. The key findings were: (1) Approximately one-half of DWI offenders eligible for the service participated, and offenders who declined to participate were more likely to endorse low-risk alcohol use. (2) The majority of those screened reported at-risk alcohol use patterns, with approximately one-quarter willing to endorse experiencing mental health symptoms at levels requiring further assessment. (3) Clients reported high ratings of usability across all domains examined, with regards to the MATTERS screening tool (ease-of-use, time, aesthetics, and comprehension). (4) There were relatively few minor technical difficulties. (5) The majority of clients reported that the information provided in the personalized feedback report was easy to comprehend, interesting, personally relevant, and likely to increase motivation to change alcohol use and to impact drinking. (6) Clients reported high levels of satisfaction with the overall service. Additionally, there were no significant differences between those receiving SBIRT at pre-trial versus at the clinic. Taken together, these findings suggest that computer-assisted SBIRT can be successfully implemented within the criminal justice system to DWI offenders soon after arrest.

Research has previously demonstrated that alcohol use disorders are highly prevalent among DWI offenders [1, 2, 30–33], yet a substantial proportion do not receive treatment, most likely the result of no health insurance and limited access to care [6]. Our treatment clinic is specifically designed to treat DWI offenders most vulnerable to unmet treatment needs (i.e., those with no private health insurance and low income) [6]. The current program evaluation demonstrated that approximately 50 percent of those eligible (i.e., those with no private health insurance and low income) participated in the SBIRT session, with the majority participating while attending their DWI pre-trial orientation session. This demonstrates that computer-assisted SBIRT, when delivered within the criminal justice system, provides access to a difficult-to-reach and underserved population.

Previous research has shown that offenders are often unwilling to report accurate levels of alcohol use or information regarding the negative consequences of their use [34, 35]. The results of the current program evaluation, however, demonstrated that a high percentage of DWI offenders reported at-risk alcohol use levels, and a substantial proportion were willing to endorse mental health symptoms at levels requiring further assessment. It is possible that such high rates of endorsement may be due to the fact that the sessions were conducted by noncriminal justice staff; however, all clients provided consent to disclose information to pre-trial services regarding their participation in the program, although this did not include the results of their assessment. An alternative explanation for such levels of endorsement may be due to the fact that a computerized screening tool was used. Research has shown previously that individuals may be more likely to report stigmatized or negative behaviors in computer-based interviewing rather than in face-to-face interviewing [36, 37]. Indeed, Lotfipour et al. [38] demonstrated increased detection of at-risk drinking when a computerized screening tool was used in comparison to a medical screening examination conducted by a nurse during an emergency department visit. Therefore, computer-assisted screening may be a more feasible method for screening at-risk behaviors in the criminal justice system.

Consistent with previous studies examining the use of computer-assisted SBIRT programs in medical settings [25, 39], the program evaluation showed that clients reported high levels of usability and satisfaction with regards to the MATTERS screening tool. We do acknowledge that there was an issue with regards to the size of the computer screen that the tool was administered on. Although only 6 percent of clients suggested that the tool could be improved by using a bigger screen, the counselor had to assist in 18 percent of assessments due to this issue, because several clients were unable to view the writing on the screen adequately. This issue, however, does not require any changes be made to the MATTERS screening tool, but does suggest that to enhance usability, display settings should be changed to increase the size of the text.

Furthermore, our findings demonstrated that although the majority of clients had prior experience with computers and reported relatively high levels of comfort with computers, 23 percent of clients reported never to have used computers. Despite this, the counselor had to assist in the completion of the MATTERS screening tool in less than 5 percent of assessments due to lack of experience with computers. Although this is promising in terms of feasibility, it could be that inexperienced clients who did not ask for assistance had a lack of understanding, which led them to give inaccurate responses. This explanation, however, seems unlikely considering that clients reported high ratings of usability in terms of ease-of-use and comprehension of both the instructions and questions and also reported that overall they found the computerized session to be extremely helpful. As such, experience with computers and levels of comprehension do not appear to be barriers to the feasibility of implementing computer-assisted SBIRT within the criminal justice system to DWI offenders.

In addition to high levels of usability and satisfaction with regards to the MATTERS screening tool, the majority of clients reported high levels of acceptability and positive perceptions with regards to the personalized feedback report. In addition to high rates of comprehension, the majority of clients also reported that the information was highly interesting, personally relevant, likely to increase their motivation to reduce alcohol, and likely to impact their own alcohol use as well as use in other DWI defendants. Prior research suggests that interventions that incorporate personalized feedback are effective in reducing alcohol use [40, 41], and although further research is required to examine the impact of this SBIRT program on alcohol use, the acceptability and perception ratings reported appear promising.

Overall, the findings of the current program evaluation demonstrate that computer-assisted SBIRT can be implemented within the criminal justice system to DWI offenders most vulnerable to unmet treatment needs. Although the SBIRT session was delivered by a counselor in our program due to being part of a larger treatment clinic providing extended services, given the usability of the MATTERS screening tool, criminal justice staff could be trained to deliver this SBIRT program soon after arrest. For example, given that the MATTERS screening tool is designed to be a self-guided program, defendants could potentially log in and complete the assessment themselves prior to meeting with a pre-trial officer. Pre-trial officers could be trained to then use the personalized feedback report to deliver the brief intervention and referral to treatment during their one-on-one interviews with the offenders at the pre-trial orientation sessions. In fact, other research has shown that brief motivational interviewing can be effectively delivered by probation officers [42].

In light of the rates of untreated alcohol problems among DWI offenders and the association between problematic patterns of alcohol use and recidivism, computer-assisted SBIRT may offer a low-cost intervention that could have significant impact on public safety. Although this program evaluation demonstrates feasibility of implementation, future research is required to examine the effectiveness of this program, with regards to clients successfully engaging in treatment and the effects it has on recidivism.
